# Neuroprotective Effects of Alpha Lipoic Acid on Haloperidol-Induced Oxidative Stress in the Rat Brain

**DOI:** 10.1186/2045-3701-1-12

**Published:** 2011-03-22

**Authors:** Joachim Perera, Joon Heng Tan, S Jeevathayaparan, Srikumar Chakravarthi, Nagaraja Haleagrahara

**Affiliations:** 1 Department of Human Biology, International Medical University, Kuala Lumpur, Malaysia; 2 Department of Pathology, International Medical University, Kuala Lumpur, Malaysia

## Abstract

Haloperidol is an antipsychotic drug that exerts its' antipsychotic effects by inhibiting dopaminergic neurons. Although the exact pathophysiology of haloperidol extrapyramidal symptoms are not known, the role of reactive oxygen species in inducing oxidative stress has been proposed as one of the mechanisms of prolonged haloperidol-induced neurotoxicity. In the present study, we evaluate the protective effect of alpha lipoic acid against haloperidol-induced oxidative stress in the rat brain. Sprague Dawley rats were divided into control, alpha lipoic acid alone (100 mg/kg p.o for 21 days), haloperidol alone (2 mg/kg i.p for 21 days), and haloperidol with alpha lipoic acid groups (for 21 days). Haloperidol treatment significantly decreased levels of the brain antioxidant enzymes super oxide dismutase and glutathione peroxidase and concurrent treatment with alpha lipoic acid significantly reversed the oxidative effects of haloperidol. Histopathological changes revealed significant haloperidol-induced damage in the cerebral cortex, internal capsule, and substantia nigra. Alpha lipoic acid significantly reduced this damage and there were very little neuronal atrophy. Areas of angiogenesis were also seen in the alpha lipoic acid-treated group. In conclusion, the study proves that alpha lipoic acid treatment significantly reduces haloperidol-induced neuronal damage.

## Background

Haloperidol (HP) is a first generation antipsychotic frequently used in the treatment of schizophrenia. It is a drug of the butyrophenone class of neuroleptics [1] and is thought to exert its antipsychotic effects by blocking dopamine, especially through D2 receptor inhibition [2]. HP has strong activity against delusions and hallucinations due to its effects on the mesolimbic pathway of the brain. Its action on the nigrostriatal pathway, on the other hand, is responsible for the side effects. The effect of long-term administration of HP on the activity of dopaminergic neurons is termed 'depolarization blockade'; the chronic effect of HP is an inactivation of dopamine neurons of the mesolimbic and nigrostriatal pathways [3]. Researchers have postulated that the 'depolarization block' of the mesolimbic pathway is responsible for the therapeutic efficacy of HP while the decreased activity of the substantia nigra gives rise to the extra-pyramidal side effects [4].

Usage of haloperidol is limited by the drug's tendency to exhibit a range of extra-pyramidal symptoms such as Parkinsonism and tardive dyskinesia [5]. Tardive dyskinesia is a serious neurological syndrome associated with the chronic use of neuroleptics. Such patients present with symptoms such as involuntary lip smacking, sucking or chewing, facial grimacing, or choreoathetoid-like movements of limbs [6]. Tardive dyskinesia may persist for months or years despite withdrawal and may be irreversible.

The exact pathophysiology of haloperidol-induced extra-pyramidal symptoms has yet to be elucidated. Oxidative stress resulting from increased production of reactive oxygen species (ROS) and a decrease in antioxidant defense mechanisms is proposed as a pathogenic mechanism. Treatment with HP causes dopaminergic receptor blockade resulting in increased dopamine turnover rate. This can lead to generation of reactive oxygen species as byproducts of its metabolism [7]. Apart from the production of free radicals, HP administration is also associated with the significant decrease in levels of the antioxidant glutathione. Previous research has also shown that coadministration of haloperidol and antioxidants such as vitamin E resulted in beneficial effect in patients with tardive dyskinesia [8].

Superoxide dismutase (SOD) and glutathione peroxidase (GPx) are important antioxidant enzymes in the body. SOD catalyses the conversion of superoxide free radical to hydrogen peroxide and water [9]. GPx continues where SOD leaves off by catalyzing the reduction of hydrogen peroxide to water at the expense of glutathione [10]. Previous studies have shown that HP reduces the activity of SOD and GPx [11] rendering the body more vulnerable to oxidative damage.

Alpha lipoic acid (ALA) is a naturally occurring compound synthesized in small quantities by most plants and animals. In humans, ALA is synthesized in the mitochondria from octanoic acid [12]. ALA functions as an important cofactor for several important enzymes. It has been shown recently that ALA also has a role in the oxidative decarboxylation of pyruvate to acetyl CoA, the step that bridges glycolysis and the Krebs cycle [13]. ALA can be found in dietary sources such as animal liver, spinach, broccoli, and tomatoes [14,15].

Huge interest has been garnered in recent times on the antioxidant properties of ALA and its reduced form dihydrolipoic acid (DHLA). Both ALA and DHLA are able to cross the blood-brain barrier and act as a redox couple with very low reduction potentials [13]. Due to these properties ALA is capable of regenerating other important antioxidants such as glutathione, vitamin C, and vitamin E [16,17]. It also has specificity for free radical quenching and metal chelating apart from regeneration of other cellular antioxidants. Furthermore, due to its action in both the membranous and aqueous phase, ALA is frequently referred to as a universal antioxidant.

## Results and Discussion

**Figure 1:**
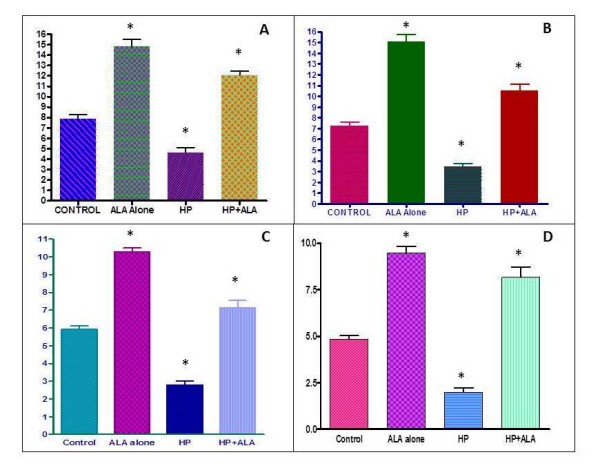
**Superoxide dismutase (SOD) and Glutathione peroxidase levels in serum and brain.** A - Serum SOD {Mean SOD Activity (U/ml)} B - Brain SOD {Mean SOD activity (U/ml)} C - Serum GPx {Mean GPx activity (nmol/min/ml)}D - Brain GPx {Mean GPx activity (nmol/min/ml)}, Values are Mean ± SEM, p < 0.05.

Figure 1A and 1B show the mean activities of SOD and figure 1C and 1D show the mean activities of GPx in the serum and brain respectively. The results were compared statistically and the mean activity of SOD and GPx was significantly higher (p < 0.01) in the ALA group than in the control group. On the other hand, the mean activity of SOD and GPx was significantly lower (p < 0.01) in the HP group than in the control group. Comparing group 3 and group 4, it was noticed that the rats that were co-administered HP and ALA showed a significantly higher (p < 0.01) increase in SOD and GPx activity in serum and brain compared to the rats that were given HP alone.

**Figure 2:**
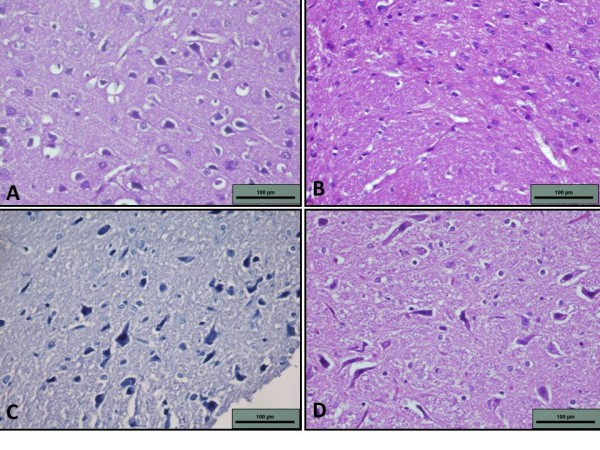
**Photomicrograph of neurons (400×, H&E stain).** A - Photomicrograph of A - control group depicting normal neurons and axons. B - ALA group showing normal parenchymal tissue with good cellular morphology and surrounding glial tissue, C - HP group showing atrophic nerve fibers and loss of prominent axons, D - ALA+HP group showing normal and healthy neurons (400×, H&E stain).

**Figure 3:**
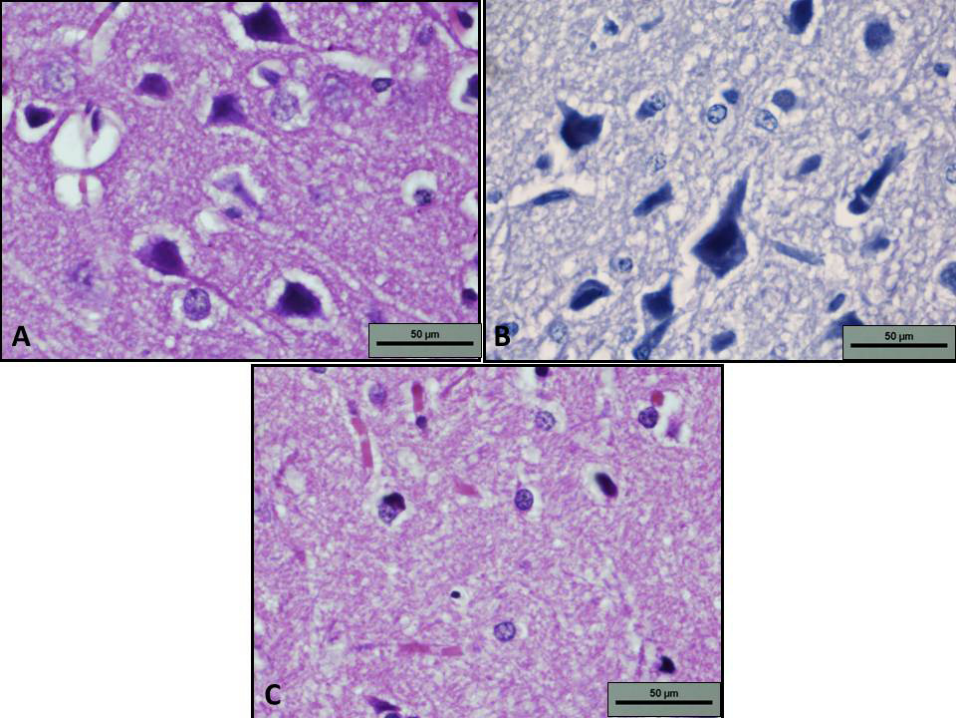
**Photomicrograph of neurons with visible axons (1000×, H&E stain).** Photomicrograph of A - control group showing normal neurons with visible axons. B - HP group showing atrophic nuclei at a higher magnification with loss of prominent axons and dendrites. C - Photomicrograph of ALA+HP group showing formation of numerous capillaries (1000×, H&E stain).

The histological study of the contol brains showed normal cellularity of the cerebral cortex and the basal ganglia. Healthy neurons were seen with prominent axons and dendrites (Figures. 2A and 3A). The histopathological findings in the ALA-treated group were similar to those in the control group (Figure 2B)

The group treated with HP showed changes that suggested morphological damage (Figures. 2C and 3B). Necrosis occurred in the cerebral cortex, internal capsule, and substantia nigra. Layers of the cerebral cortex were obliterated and replaced with zones of fibromyxoid stroma. Fibrosis was also extensive in the internal capsule and substantia nigra. When examined with higher magnification, neurons were found to be atrophic and there was loss of prominent axons and dendrites. In the group treated with both ALA and HP, a small amount of necrosis occurred in the substantia nigra but the intensity of damage was minimal compared to the group treated with HP alone. In most rats, the cerebral cortex and internal capsule were spared. Areas of angiogenesis were also noted, with sprouting of numerous capillaries and arterioles within areas of necrosis. Atrophic neurons were infrequent and the majority of neurons were healthy with visible axons and dendrites (Figures. 2D and 3C).

Rats treated with HP showed a significant decrease in mean SOD and GPx activity. These results supported the hypothesis that HP induces oxidative stress. SOD catalyzes the dismutation of superoxide radicals to form hydrogen peroxide, which in turn is decomposed to water and oxygen by GPx. These enzymes work together to protect the body from free radicals. The reduced activity of SOD and GPx in rats treated with HP increases the vulnerability to the effects of free radicals. Oxidative stress is compounded by the generation of free radicals due to the action of HP. HP blockade of dopamine receptors increases dopamine turnover, which in turn leads to the increased production of hydrogen peroxide. The decrease in enzymatic activities of SOD and GPx is possibly due to HP decreasing the genetic expression of these enzymes [18]. On the other hand, the activity of these enzymes was brought back to normal levels with co-administration of ALA. This result probably can be explained by the effect of ALA on nerve growth factor (NGF). NGF provides the expression of SOD gene and there is a possibility that ALA enhances NGF-induced regulation of SOD gene. This could be a factor leading to an increase in SOD activity [19]; GPx activity too was also restored to a normal level by the co-administration of ALA. GPx catalyzes the reduction of hydrogen peroxide to water and oxygen at the expense of glutathione (GSH). Therefore the increase in GPx activity indicates that more oxidized glutathione (GSSH) is reduced to GSH. This recycling of glutathione is hypothesized to be due to ALA.

The relationship between the results of the histopathological and biochemical analyses in our study is striking. The increased vulnerability of the rat brain to oxidative stress as shown by decreased antioxidant enzymes was mirrored in histopathological findings. Degenerative changes were apparent in the striatum, substantia nigra, and internal capsule in rats treated with HP. The resulting photomicrographs revealed massive areas of necrosis, fibrosis, and atrophic neurons. These findings are in accordance with previous studies that showed nigral pathology upon HP administration [20,21]. Pathological changes also strengthen the free radical hypothesis of HP. The inhibitory action of HP on D_2 _receptors causes an increased turnover in dopamine which then leads to the formation of hydrogen peroxide and other free radicals. The increase in free radical formation, coupled with decreased SOD and GPx enzyme activity in the brain as established in our study is possibly the basis for oxidative damage to the brain. The degenerative changes were not confined to the basal ganglia and internal capsule but affected the cerebral cortex as well indicating the extent of the HP-induced oxidative damage.

When ALA was co-administered with HP, the cerebral cortex and internal capsule were spared of degenerative changes. There was normal cellular morphology and each layer of the cerebral cortex was intact but necrosis and atrophic neurons still were found in the basal ganglia. Nevertheless, necrotic and atrophic changes were smaller when ALA was co-administered with HP than when HP was administered alone. Thus, ALA may have played a protective role in HP-induced oxidative damage in the brain. The sprouting of numerous capillaries that was seen in necrotic areas of the brain signifying neovascularisation suggests that ALA both protects the brain from oxidative damage and facilitates healing.

The neuroprotective effects of ALA demonstrated in this study can be explained by its antioxidant properties. ALA is reduced to DHLA in the cells, forming a redox couple. ALA and DHLA prevent oxidative damage by interacting with ROS. ALA and DHLA are able to scavenge free radicals such as singlet oxygen and hydroxyl radicals. They also chelate metal ions which are involved in ROS formation. In the event that SOD and GPx activities were affected when the rats were treated with HP, ALA probably compensates for SOD and GPx action. Moreover, ALA is also known to regenerate other antioxidants such as glutathione, vitamin E, vitamin C, and coenzyme Q10. The newly reduced glutathione, vitamin C, and coenzyme Q also regenerate oxidized vitamin E, forming an antioxidant network that protects the body from HP-induced oxidative damage.

The present study showed that HP causes oxidative stress. This conclusion was based on the findings that administration of HP significantly decreased SOD and GPx activity in rat brain and serum. Histopathological studies showed the deleterious effect of HP on the rat brain as massive necrosis, fibrosis, and atrophic neurons. The histopathological changes correlated with the biochemical assay results.

We found that rats treated with ALA showed a much greater increase in body weight. In addition, rats treated with ALA also showed a significant increase in brain ACh concentration. Our other objective was to investigate the effects of ALA on HP-induced alterations towards the antioxidant defense system in the rat brain. The administration of ALA to HP-treated rats, restored the activity of SOD and GPx in both serum and brain and demonstrated a distinct protective effect in histopathological studies. The significant increase in antioxidant activity coupled with the histological evidence, leads to the conclusion that ALA reduces HP-induced oxidative damage in rat brain.

## Methods

A total of 32 male Sprague-Dawley rats (180-250 g body weight) were used in this study. The rats were housed under standard laboratory conditions (temperature of 24 ± 3°C, humidity 40-60%) with 12 hour light and dark cycles. Food and water were supplied *ad libitum *prior to the start of the experiment. All the experiments were carried out according to the standard guidelines for animal experiments and Institutional Ethics Committee has approved this research project.

Each drug used in this experiment was purchased in the powder form (Sigma Aldrich Co. USA); consequently, each drug was dissolved before administration to rats. Rats were weighed daily. Drug solutions were given at 1 ml/200 g body weight. ALA solution was given oral gavage using a stainless steel oral feeding tube. Intraperitoneal injections of HP were administered using a 1.0 mL syringe with a 21 gauge needle to prevent damage to the internal organs of rats.

On the 22^nd ^day of the experiment, the rats were anaesthetized with ether. A blood sample was collected by cardiac puncture. Blood was transferred into 5 ml tubes, which were centrifuged using Sigma 2-16 KC Refrigerated Centrifuge at 3000 rpm for 10 minutes at 4°C. The serum was extracted using a 1 ml syringe and stored at -20°C until further analysis.

Brain was dissected out and photographed using a digital camera, for gross inspection. Brain was sectioned into two halves. One half of the brain was kept in 50 ml tube filled with 10% formalin for a month before processing for histopathological studies. The other half of the brain was transferred into 15 ml Falcon tubes filled with phosphate buffer solution and stored at -20°C. Brain samples were thawed and homogenized in ice to ensure the viability of proteins using a Duall, all-glass, tissue grinder. The homogenate was centrifuged using Sigma 2-16 KC Refrigerated Centrifuge at 6000 rpm for 20 minutes at 4°C. The samples were maintained at -20°C before performing biochemical assays. From the brain homogenate samples, superoxide dismutase (SOD) and glutathione peroxidase (GPx) were assayed using ELISA kits (Cayman Chemicals and Pierce Biotechnology, USA).

Super oxide dismutase assay kit utilizes a tetrazolium salt for the detection of superoxide radicals (O2-) generated by xanthine oxidase and hypoxanthine. One unit of SOD is defined as the amount of enzyme necessary to exhibit 50% dismutation of the superoxide radical. Oxidation rate of tetrazolium salt to formazan dye by O2 - is inversely proportional to the endogenous activity of SOD. The formazan dye stains the wells and its staining intensity was detected by absorbance spectrophotometry at 450 nm using a plate reader. Superoxide dismutase levels were determined from a standard curve and expressed as U/mg of protein [22,23].

Glutathione peroxidase assay kit measures GPx levels indirectly by a coupled reaction with glutathione reductase. Oxidized glutathione, produced upon reduction of an organic hydroperoxide by GPx, is recycled to its reduced state by glutathione reductase and NADPH. The oxidation of NADPH to NADP+ is accompanied by a decrease in absorbance at 340 nm. The rate of decrease in the absorbance at 340 was directly proportional to the GPx levels in the sample. Glutathione peroxidase enzyme levels were expressed as nmol/mg of protein [22,24].

Statistical analysis was done using Statistical Package for Social Sciences (SPSS) version 15.0. Data obtained were expressed as mean ± standard deviation (SD). Analysis of variance (ANOVA) followed by multiple range test was used to determine significant differences. Differences at p < 0.05 were considered to be statistically significant.

## Competing interests

The authors declare that they have no competing interests.

## Authors' contributions

JP conceived of the study and coordination and helped to draft the manuscript. JHT carried out the experimental work, done the analysis of the data and prepared the results. JS performed the statistical analysis. SC carried out the histopathological analysis. NH participated in the design of the study and contributed to analysis of the results. All authors read and approved the final manuscript.
